# Carboxyl-Functionalized Polymeric Microspheres Prepared by One-Stage Photoinitiated RAFT Dispersion Polymerization

**DOI:** 10.3390/polym9120681

**Published:** 2017-12-06

**Authors:** Jianbo Tan, Xueliang Li, Jun He, Qin Xu, Yuxuan Zhang, Xiaocong Dai, Liangliang Yu, Ruiming Zeng, Li Zhang

**Affiliations:** 1 Department of Polymeric Materials and Engineering, School of Materials and Energy, Guangdong University of Technology, Guangzhou 510006, China; a2687575196@163.com (X.L.); qq524358939@163.com (J.H.); xq13246857614@163.com (Q.X.); 15918627483@163.com (Y.Z.); dai251798160@163.com (X.D.); liangliangyu1230@163.com (L.Y.); ray18813293416@163.com (R.Z.); 2 Guangdong Provincial Key Laboratory of Functional Soft Condensed Matter, Guangzhou 510006, China

**Keywords:** carboxyl-functionalized microspheres, RAFT polymerization, photoinitiated dispersion polymerization

## Abstract

Herein, we report a photoinitiated reversible addition-fragmentation chain transfer (RAFT) dispersion copolymerization of methyl methacrylate (MMA) and methyl methacrylic (MAA) for the preparation of highly monodisperse carboxyl-functionalized polymeric microspheres. High rates of polymerization were observed, with more than 90% particle yields being achieved within 3 h of UV irradiation. Effects of reaction parameters (e.g., MAA concentration, RAFT agent concentration, photoinitiator concentration, and solvent composition) were studied in detail, and highly monodisperse polymeric microspheres were obtained in most cases. Finally, silver (Ag) composite microspheres were prepared by in situ reduction of AgNO_3_ using the carboxyl-functionalized polymeric microspheres as the template. The obtained Ag composite microspheres were able to catalyze the reduction of methylene blue (MB) with NaBH_4_ as a reductant.

## 1. Introduction

Micron-sized polymeric microspheres have attracted much attention due to their broad applications in catalysis, molecular separation, molecular imprinting, Pickering emulsion, and biomedical analysis [1,2,3,4,5,6]. These polymeric microspheres are most commonly prepared using the seeded swelling method developed by Ugelstad [7] or the seeded emulsion polymerization developed by Vanderhoff [8]. However, these methods are complex and difficult to implement on a large scale.

Dispersion polymerization is a very attractive one-pot strategy for the preparation of micron-sized polymeric microspheres with narrow particle size distributions [9]. For dispersion polymerization, all reagents including monomers, stabilizers, and initiators are dissolved in the reaction medium. As the polymerization proceeds, polymers grow to a critical length and then precipitate from the reaction medium to form irregular nuclei. The formed nuclei are subsequently stabilized by stabilizers, and then grow in parallel to form polymeric microspheres with narrow particle size distributions.

The carboxyl group is an important functional group for polymeric microspheres that can be used for composite materials, as well as biomedical or biodiagnostic applications [10,11,12,13]. In dispersion polymerization, the introduction of carboxyl groups to polymeric microspheres can be easily achieved by copolymerization with carboxyl-containing comonomers such as acrylic acid (AA) or methacrylic acid (MAA) [14,15]. However, the nucleation stage of traditional dispersion polymerization is very sensitive to the presence of functional regents, which usually leads to the formation of polydisperse particles, and even coagulation. The Winnik group [14,16,17,18] developed a two-stage dispersion polymerization method for the synthesis of monodisperse functional polymeric microspheres (e.g., carboxyl-functionalized, cross-linked, reactive) with functional reagents added in the particle growth stage. For example, Song et al. [14] synthesized carboxyl-functionalized polystyrene microspheres using the two-stage dispersion polymerization with 6 wt % AA added in the particle growth stage. Very recently, Tan et al. [19,20,21] developed a novel one-stage dispersion polymerization formulation named photoinitiated RAFT dispersion polymerization (RAFT = reversible addition-fragmentation chain transfer). For photoinitiated dispersion polymerization, the nucleation period is too short to obtain monodisperse polymeric microspheres, which can be attributed to the inefficient stabilization of stabilizers on the nuclei. The introduction of the RAFT technique into photoinitiated dispersion polymerization makes the nucleation stage longer, allowing the formation of uniform microspheres. Moreover, the presence of functional reagents did not affect the uniformity of polymeric microspheres.

In this paper, we focused on the preparation of monodisperse carboxyl-functionalized poly(methyl methacrylate) (PMMA) microspheres via photoinitiated RAFT dispersion copolymerization of MMA and MAA. The concentration of MAA can be up to 10 wt %. The effects of reaction conditions were also investigated in detail. The carboxyl-functionalized polymeric microspheres prepared by the photointiated RAFT dispersion polymerization can be employed as templates for the synthesis of silver (Ag) composite microspheres.

## 2. Experimental Section

### 2.1. Materials

Poly(*N*-vinylpyrrolidone) (PVP, *M*_n_ = 40,000 g/mol, Aladdin), 2-hydroxy-2-methylpropiophenone (HMPP, Ciba Company, Basel, Switzerland), methacrylic acid (MAA, Aladdin, Shanghai, China), silver nitrate (AgNO_3_, Aladdin, Shanghai, China), sodium borohydride (NaBH_4_, Aladdin, Shanghai, China), and methylene blue (MB, Aladdin, Shanghai, China) were used without further purification. Methyl methacrylate (MMA, Aladdin, Shanghai, China) was purified by passing through a basic alumina oxide (Aladdin, Shanghai, China) column prior to storage at 4 °C. *S*,*S*′-bis(α,α′-dimethyl-α″-aceticacid) trithiocarbonate (BDMAT) was synthesized according to a published procedure [22].

### 2.2. Characterization

*Scanning electron microscopy (SEM).* SEM observations were carried out on a Hitachi S3400N scanning electron microscopy (SEM) (Tokyo, Japan) operated at 10 kV. Samples were diluted with water and dropped on a mica film. The samples were dried at room temperature and gold-coated prior to the SEM measurement. SEM images were analyzed by using the software program named ImageJ (NIH, Bethesda, MD, USA). The average diameter (*d*_n_) and coefficient of variation of diameter (*CV*_d_) were calculated according to the equations:

(1) $$d_{n}=\sum_{i=1}^{n}n_{i}d_{i}/n,CV_{d}=\sqrt{\frac{\sum_{i=1}^{n}{\left(d_{i}-d_{n}\right)}^{2}}{n-1}}/d_{n}$$

*Transmission electron microscope* (TEM). TEM observations were carried out on a FEI Technai G2 Spirit instrument (Hillsboro, OR, USA) operated at 120 kV. Samples were dispersed in ethanol/water, and a drop of the solution was placed on copper grids and allowed to dry at room temperature.

*UV-vis Spectroscopy.* The UV-Visible spectra were recorded with a 1.0 cm quartz cuvette using a UV2450 spectrometer (Shimadzu, Kyoto, Japan).

*^1^H NMR Spectroscopy.* ^1^H NMR spectra were recorded in CDCl_3_ using a Bruker Advance III 400 MHz NMR spectrometer (Billerica, MA, USA) at 25 °C.

*Fourier transform infrared (FTIR) spectroscopy.* Each sample was mixed with KBr and then compressed into a pallet. FTIR spectra were measured using a Nicolet 380 spectrometer (Thermo Nicolet Corporation, Madison, WI, USA).

Gel permeation chromatography (GPC) measurement. The molecular weight and molecular weight distribution of polymeric microspheres were measured by GPC using a Waters 1515 GPC instrument (Milford, CT, USA) with tetrahydrofuran (THF) as the mobile phase and Waters styragel HR1, HR4 columns (Milford, CT, USA). The flow rate of THF was kept at 1.0 mL/min. A series of poly(methyl methacrylate) polymers with narrow molecular weight distributions were used as the standard to calibrate the apparatus.

### 2.3. Photoinitiated RAFT Dispersion Polymerization of MMA and MAA

In a typical experiment (2 wt % MAA): MMA (2.0 g, 10 wt % relative to the reaction mixture), MAA (0.04 g, 2 wt % relative to MMA), BDMAT (0.01 g, 0.5 wt % relative to MMA), PVP (0.30 g, 15 wt % relative to MMA), and HMPP (0.06 g, 3 wt % relative to MMA) were dissolved in an ethanol/water (7.2 g/10.8 g) mixture. The reaction mixture was purged with nitrogen for 15 min, sealed, and then irradiated with a LED lamp (365 nm, 0.8 mW/cm^2^) at room temperature for 3 h. The sample was purified by three cycles of centrifugation-redispersion in ethanol/water (40/60, *w/w*). The obtained product was dried in a vacuum oven at 45 °C overnight and weighed to calculate the yield.

### 2.4. Synthesis of Ag Composite Microspheres

MMA (2.0 g, 10 wt % relative to the reaction mixture), MAA (0.2 g, 10 wt % relative to MMA), PVP (0.30 g, 15 wt % relative to MMA), BDMAT (0.01 g, 0.5 wt % relative to MMA), and HMPP (0.06 g, 3 wt % relative to MMA) were dissolved in an ethanol/water (7.2 g/10.8 g) mixture. The reaction mixture was purged with nitrogen for 15 min, sealed, and then irradiated with a LED lamp (365 nm, 0.8 mW/cm^2^) at room temperature for 3 h. Subsequently, a certain amount of AgNO_3_ (50 mg) was added to the reaction mixture and irradiated for another 3 h. The sample was purified by centrifugation, rinsed with ethanol/water (40/60, *w/w*), and centrifuged repeatedly.

### 2.5. Catalytic Reduction of Methylene Blue (MB)

A dispersion of Ag composite microspheres was mixed with an aqueous solution of MB (0.01 wt %, 10 mL). Then, an aqueous solution of NaBH_4_ (0.65 M, 1 mL) was added to the mixture. The catalytic property of PMMA/Ag composite microspheres was measured by monitoring the variation in the optical properties of the dye using a UV-Vis spectrometer.

## 3. Results and Discussion

### 3.1. Photoinitiated RAFT Dispersion Copolymerization of MMA and MAA

It should be noted that a new type of RAFT dispersion polymerization has been developed over the past 10 years. However, this RAFT dispersion polymerization just focuses on the synthesis of polymer nano-objects with different morphologies rather than monodisperse polymeric microspheres [23,24,25,26,27,28,29,30,31,32]. Herein, we first carried out photoinitiated RAFT dispersion polymerization of MMA in an ethanol/water (40/60, *w/w*) mixture in the absence of MAA. 2-Hydroxy-2-methypropiophenone (HMPP) was employed as the photoinitiator. Poly(*N*-vinylpyrrolidone) (PVP), the most common employed stabilizer in dispersion polymerization, was employed as the stabilizer in this paper. A carboxyl functional RAFT agent, *S*,*S*′-bis(α,α′-dimethyl-α″-aceticacid) trithiocarbonate (BDMAT), was used to control the polymerization. It should be noted that HMPP decomposes rapidly upon the exposure to UV irradiation, and a UV-LED lamp was employed as the light source.

**Figure 1:**
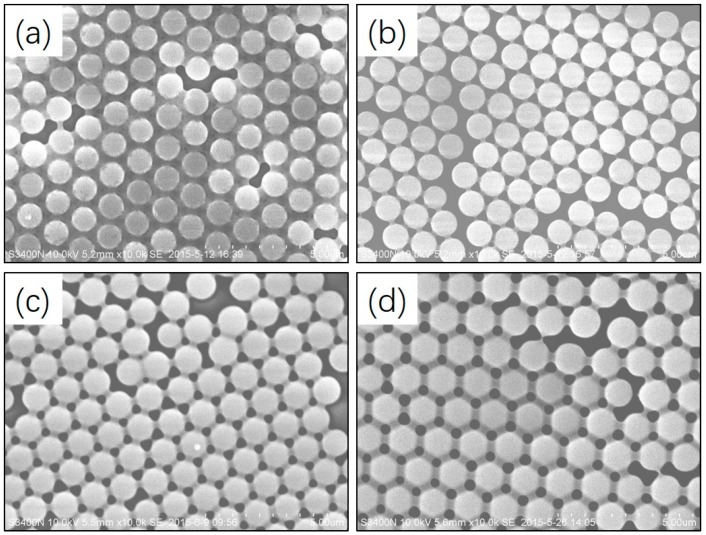
Scanning electron microscopy (SEM) images of polymeric microspheres prepared by photoinitiated reversible addition-fragmentation chain transfer (RAFT) dispersion polymerization of methyl methacrylate (MMA) with different concentrations of methyl methacrylic (MAA) added at the beginning of the reaction: (**a**) 0 wt %, (**b**) 2 wt %, (**c**) 6 wt %, (**d**) 10 wt %.

Figure 1 shows SEM images of polymeric microspheres prepared by photoinitiated RAFT dispersion polymerization of MMA with different concentrations of MAA. Table 1 shows the employed reaction conditions and particle sizes. As a reference, we note that in the absence of MAA, uniform particles with *d*_n_ = 0.92 µm, *CV*_d_ = 3.1% were obtained (Figure 1a). Polymeric microspheres with narrow particle size distributions were obtained with the concentrations of MAA ranging from 2 to 10 wt %. When the concentration of MAA was 2 wt %, the particle size was increased slightly to 0.94 µm with a narrow particle size distribution (*CV*_d_ = 2.5%). When the concentration of MAA was further increased to 10 wt %, uniform particles with similar size were obtained (*d*_n_ = 1.09 µm, *CV*_d_ = 2.4%). The main conclusion from these experiments is that the uniformity of particles is robust to the amount of MAA. The samples were also characterized by IR spectroscopy (see Figure S1 in the supplementary materials) and ^1^H NMR spectroscopy (see Figure S2 in the supplementary materials). However, due to the similar structure of MMA and MAA, it was impossible to say if the structure difference increased the MAA content. THF GPC was also employed to characterize the samples (see Table S1 in the supplementary materials). The molecular weight distributions were broad in all cases, which can be ascribed to the high (photointiator)/(RAFT agent) ratio (>2.5) employed in the present case.

**Figure 2:**
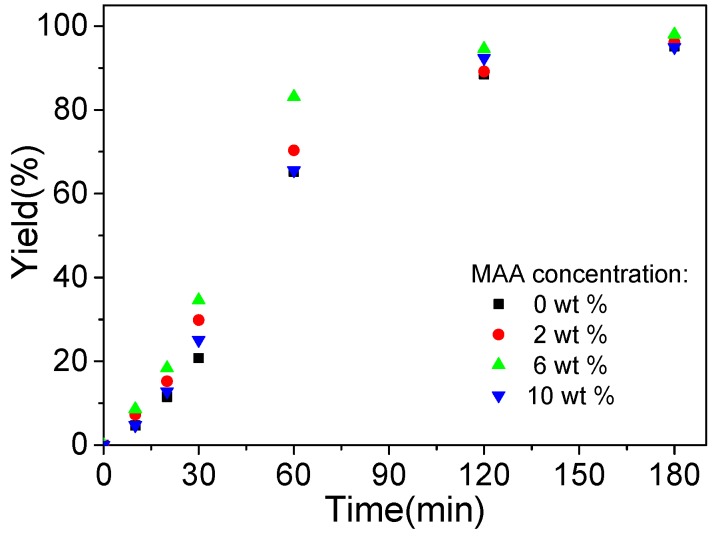
Plots of particle yield versus ultra violet (UV) irradiation time of polymeric microspheres prepared by photoinitiated RAFT dispersion polymerization with different concentrations of MAA.

**Figure 3:**
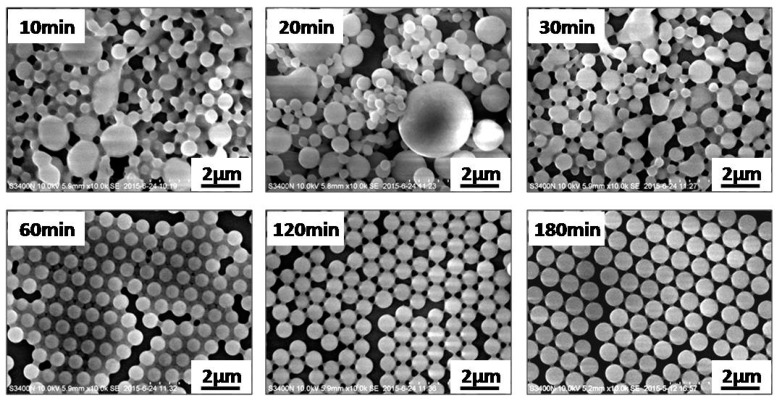
SEM images of polymeric microspheres prepared by photoinitiated RAFT dispersion copolymerization of MMA and MAA (in the presence of 2 wt % MAA) at different irradiation times.

To further investigate the formation process of the polymeric microspheres prepared by photoinitiated RAFT dispersion polymerization of MMA in the presence of different amounts of MAA, we followed the evolution of particle yield of irradiation time, as shown in Figure 2. Similar kinetics were observed in these cases, with high particle yields (>90%) being achieved within 3 h of UV irradiation. These results further confirm that the effect of MAA on the photoinitiated RAFT dispersion polymerization can be ignored. Samples withdrawn at different irradiation times were diluted with an ethanol/water (40/60, *w/w*) mixture and then characterized by SEM. Figure 3 shows SEM images of polymeric microspheres prepared by photoinitiated RAFT dispersion copolymerization of MMA and MAA (2 wt %) at different irradiation times. During the early stage (10–30 min), small particles and some extremely large particles coexisted. As the polymerization proceeded (60 min), the large spheres disappeared and then uniformed spheres were formed. As the polymerization further proceeded, the particle size grew in parallel. The formation of large particles during the nucleation stage can be attributed to the aggregation of low molecular polymer chains, which is similar to that of photoinitiated RAFT dispersion polymerization in the absence of MAA [19].

Having control over the particle size and uniformity of polymeric microspheres is particularly important for their applications [33,34]. Therefore, it is important to investigate the effect of reaction conditions on particle size and uniformity, especially for a new dispersion polymerization system. In the follow sections, we investigated the effect of RAFT agent concentration, stabilizer concentration, photoinitiator concentration, and solvent composition on the formation of carboxyl-functionalized polymeric microspheres via the one-stage photoinitiated RAFT dispersion polymerization.

### 3.2. Effect of RAFT Agent Concentration

**Figure 4:**
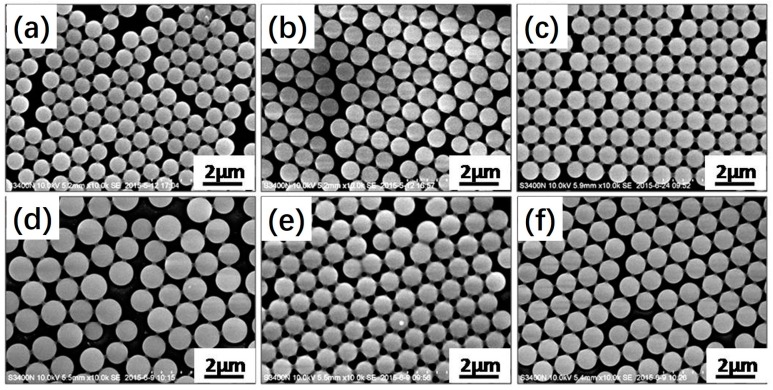
SEM images of polymeric microspheres prepared by photoinitiated RAFT dispersion polymerization of MMA at different *S*,*S*′-bis(α,α′-dimethyl-α″-aceticacid) trithiocarbonate (BDMAT) concentrations with either (**a**–**c**) 2 or (**d**–**f**) 6 wt % MAA: (**a**,**d**) 0.25 wt % BDMAT, (**b**,**e**) 0.5 wt % BDMAT, (**c**,**f**) 0.75 wt% BDMAT.

For photoinitiated RAFT dispersion polymerization, the RAFT agent plays a crucial role in obtaining uniform polymeric microspheres [20]. Figure 4 shows SEM images of polymeric microspheres prepared by photoinitiated RAFT dispersion polymerization at different BDMAT concentrations with either 2 or 6 wt % MAA. Table 2 shows the employed reaction conditions and particle sizes. When the concentration of MAA was 2 wt %, uniform polymeric microspheres were formed with 0.5 or 0.75 wt % BDMAT. When the concentration of BDMAT was decreased to 0.25 wt %, the particles were similar in size, with a somewhat broader particle size distribution (*d*_n_ = 0.95 µm, *CV*_d_ = 4.2%). Similar results were also observed when the concentration of MAA was 6 wt %. A broad particle size distribution (*CV*_d_ = 6.5%) was observed when the BDMAT concentration was 0.25 wt %. These results indicate that the RAFT agent is important for obtaining carboxyl-functionalized polymeric microspheres by photoinitiated RAFT dispersion copolymerization of MMA and MAA. Since MAA is a solvophilic comonomer, it is more likely to polymerize in the reaction medium rather than in the monomer-swollen particles. The RAFT agent allows the relatively uniform distribution of MAA in the polymer chains, which can weaken the negative effect of MAA on the photoinitiated RAFT dispersion polymerization [35]. As a control experiment, photoinitiated dispersion copolymerization of MMA and MAA (6 wt %) in the absence of BDMAT was also carried out. However, the reaction became unstable, with only precipitates being obtained upon the exposure of UV light.

### 3.3. Effect of Photoinitiator Concentration

Typically, a moderate concentration of initiator is important for dispersion polymerization to prepare uniform polymeric microspheres with high yields. When the concentration of initiator is too low, low particle yields are usually achieved. In contrast, when the concentration of initiator is too high, polydisperse particles are usually formed [36]. Therefore, it is important to investigate the effect of photoinitiator concentration on the formation of carboxyl-functionalized polymeric microspheres. 

**Figure 5:**
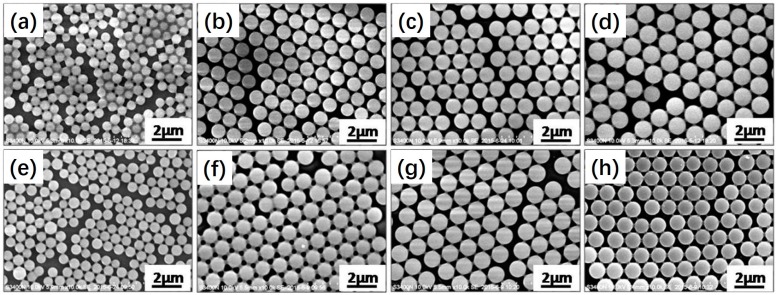
SEM images of polymeric microspheres prepared by photoinitiated RAFT dispersion polymerization of MMA at different HMPP concentrations (1–9 wt %) with either (**a**–**d**) 2 or (**e**–**h**) 6 wt % MAA: (**a**,**e**) 1 wt % HMPP, (**b**,**f**) 3 wt % HMPP, (**c**,**g**) 6 wt % HMPP, (**d**,**h**) 9 wt % HMPP.

In the present work, HMPP was employed as the photoinitiator due to its fast decomposition behavior via the exposure of UV light. Figure 5 shows SEM images of polymeric microspheres prepared by photoinitiated RAFT dispersion polymerization of MMA at different HMPP concentrations (1–9 wt %) with either 2 or 6 wt % MAA. Highly uniform polymeric microspheres were obtained in all cases, even the concentration of HMPP was up to 9 wt %. This is quite different from traditional dispersion polymerization, in which the concentration of initiator has a significant effect on the uniformity of polymeric microspheres. In traditional dispersion polymerization, a higher concentration of initiator leads to the generation of a large amount of low molecular oligomeric chains, which is prone to stay in the medium. Therefore, secondly nucleation is usually observed [36]. The insensitivity of photoinitiated RAFT dispersion polymerization to the concentration of photoinitiator may be attributed to the unique nucleation stage [19]. The nucleation stage is mainly affected by the RAFT process. Therefore, the negative effect of photoinitiator concentration on the particles can be greatly suppressed. On the other hand, these results suggest that one can tune the rate of polymerization by changing the concentration of photoinitiator without disturbing the particle size distributions.

### 3.4. Effect of Stabilizer Concentration

The main difference between dispersion polymerization and precipitation polymerization is that stabilizer is required for obtaining colloidally stable particles in dispersion polymerization. PVP is one of the most commonly used stabilizers of dispersion polymerization that stabilizes polymeric particles via physical absorption and chemical grafting [36,37]. 

**Figure 6:**
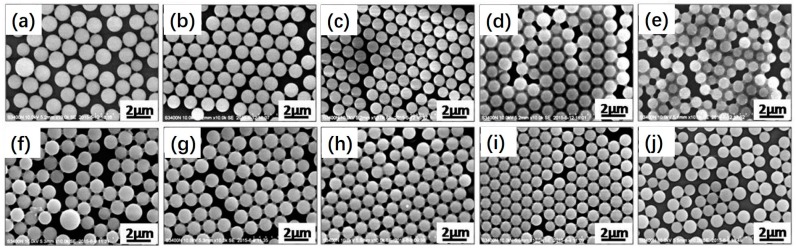
SEM images of polymeric microspheres prepared by photoinitiated RAFT dispersion polymerization of MMA at different Poly(*N*-vinylpyrrolidone) (PVP) concentrations (5–25 wt %) with either (**a**–**e**) 2 or (**f**–**j**) 6 wt % MAA: (**a**,**f**) 5 wt % PVP, (**b**,**g**) 10 wt % PVP, (**c**,**h**) 15 wt % PVP, (**d**,**i**) 20 wt %, (**e**,**j**) 25 wt %.

Figure 6 shows SEM images of polymeric microspheres prepared by photoinitiated RAFT dispersion polymerization of MMA at different PVP concentrations (5–25 wt %) with either 2 or 6 wt % MAA. Polymeric microspheres with broad particle size distributions were formed when the concentration of PVP was 5 wt %. This can be ascribed to the aggregation of growing particles, which were not stabilized sufficiently. In contrast, monodisperse polymeric microspheres were obtained, with the concentration of PVP ranging from 10 to 20 wt %. This can be attributed to the sufficient stabilization of particles when the concentration of PVP is high enough. Further increasing the concentration of PVP led to smaller particles with broad particles size distributions, which is commonly observed in traditional dispersion polymerization. These results suggest that a moderate amount of PVP is important for preparing uniform carboxyl-functionalized polymeric microspheres via photoinitiated RAFT dispersion copolymerization of MMA and MAA.

### 3.5. Effect of Solvent Composition

In dispersion polymerization, monomers are required to dissolve in the reaction medium, while the resulting polymers are not soluble. Therefore, the solvent composition should have a significant effect on the particle morphologies.

**Figure 7:**
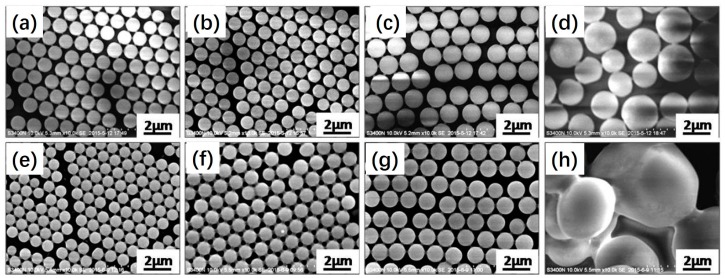
SEM images of polymeric microspheres prepared by photoinitiated RAFT dispersion polymerization at different ethanol/water ratios (*w/w*) with (**a**–**d**) 2 or (**e**–**h**) 6 wt % MAA: (**a**,**e**) 35/65, (**b**,**f**) 40/60, (**c**,**g**) 45/55, (**d**,**h**) 50/50.

Figure 7 shows SEM images of polymeric microspheres prepared by photoinitiated RAFT dispersion polymerization at different ethanol/water ratios (*w/w*) with 2 or 6 wt % MAA. In both cases, highly monodisperse polymeric microspheres were formed when the ethanol/water ratio was in the range of 35/65 to 45/55. Increasing the ethanol content led to the formation of larger particles. This can be attributed to an increase in the solubility of *P*(MMA–*co*–MAA) in the reaction mixture with increasing ethanol content, resulting in a decrease in the number of nuclei and thus bigger particles. Further increasing the ethanol/water ratio to 50/50 led to the formation of larger particles with broad particles size distributions.

### 3.6. Quantification of Carboxyl Groups on the Particle Surface

For carboxyl-functionalized polymeric microspheres, the amount of carboxyl groups on the particle surface is important for further applications. In the case of photoinitiated RAFT dispersion copolymerization of MMA and MAA, a certain amount of MAA was embedded into the particles. In this section, we intended to quantify the amount of carboxyl groups on the particle surface by titration.

**Figure 8:**
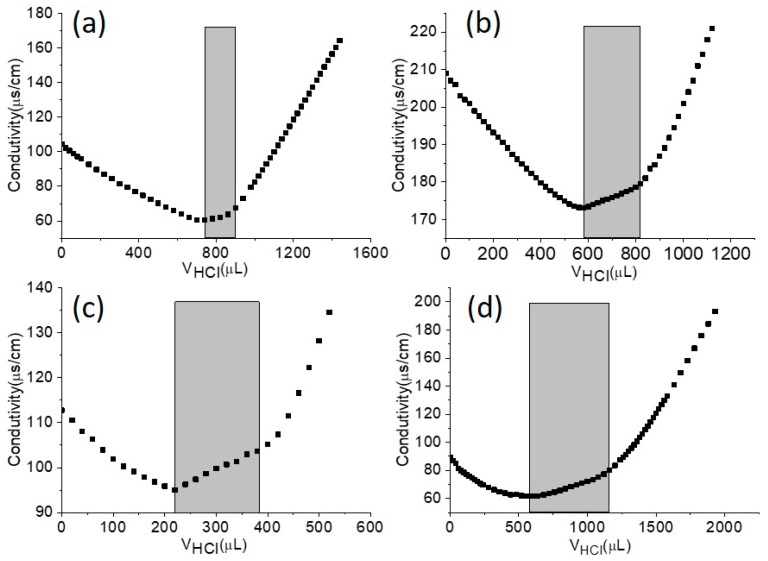
Conductometric titration curves of polymeric microspheres prepared by photoinitiated RAFT dispersion polymerization with different MAA concentrations (0–10 wt %): (**a**) 0 wt %, (**b**) 2 wt %, (**c**) 6 wt %, (**d**) 10 wt %.

It should be noted that the titration was carried out in air. To eliminate the effect of carbon dioxide in air, a blank experiment (distilled water) was also conducted in the absence of particles. Figure 8 shows conductometric titration curves of polymeric microspheres prepared by photoinitiated RAFT dispersion polymerization of MMA with different amounts of MAA (0–10 wt %). The mean number of carboxyl groups per particle can be calculate based on the follow equations:

(2) $$N=\frac{m\cdot w\%}{\rho \cdot \frac{4}{3}\pi {\left(\frac{d_{n}}{2}\right)}^{3}}$$

(3) $$N_{-\mathrm{COOH}}=\frac{C_{\mathrm{HCl}}\cdot (V_{\mathrm{HCl}}-V_{0})}{N}\cdot N_{A}$$

where *N* is the number of polymeric microspheres, *m* is the weight of dispersion, *w*% is solids content of dispersion, *ρ* is the density of PMMA (1.18 g/cm^3^), *d*_n_ is the average diameter of the polymeric microspheres, *C*_HCl_ is concentration of HCl used for the titration, *V*_HCl_ is the volume of HCl used for the titration, *V*_0_ is the volume of HCl used for the titration of distilled water, and *N*_A_ is the Avogadro constant (6.02 × 10^23^).

A small number of carboxyl groups was detected in the absence of MAA (1.2 × 10^6^) (see Table 3). This can be attributed to the utilization of BDMAT, since BDMAT is a carboxyl functional RAFT agent. Increasing the amount of MAA led to a larger number of carboxyl groups on the particle surface. For example, the number of carboxyl groups per particle was 4.1 × 10^6^ at 2 wt % MAA, while the number of carboxyl groups per particle was 1.6 × 10^7^ at 10 wt % MAA. These results suggest that one may be able to control the number of carboxyl groups on the particle surface by just changing the amount of MAA.

### 3.7. Synthesis of Organic/Inorganic Composite Microspheres

As described above, a certain number of carboxyl groups were introduced to the particle surface via the photoinitiated RAFT dispersion copolymerization of MMA and MAA. The presence of carboxyl groups on the particle surface can be used for further modification.

**Figure 9:**
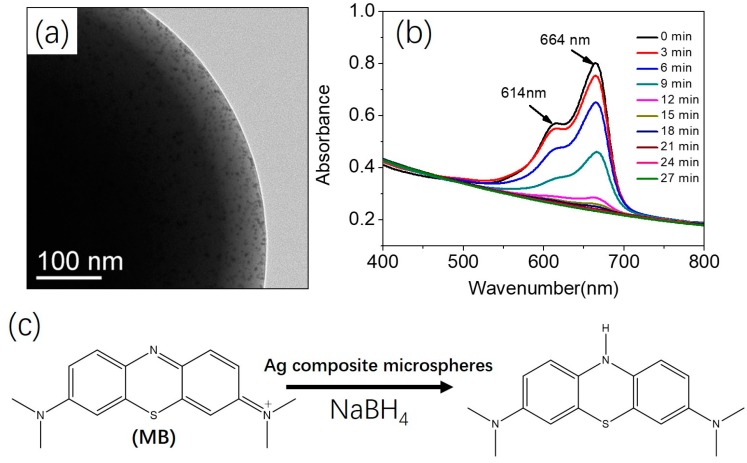
(**a**) TEM image of Ag composite microspheres, (**b**) UV-Vis absorption spectra for the catalytic reduction of methylene blue in water at room temperature using Ag composite microspheres as the catalyst, and (**c**) the reduction of methylene blue (MB) with NaBH_4_ in the presence of Ag composite microspheres.

Organic/inorganic composite microspheres with narrow particle size distributions have attracted considerable attention due to the broad applications in catalysis, energy storage, and water treatment [12,38,39,40]. As a proof-of-concept experiment, Ag composite microspheres were prepared by in situ reduction of AgNO_3_ using the carboxyl-functionalized polymeric microspheres as the template. Carboxyl-functionalized polymeric microspheres were first prepared by photoinitiated RAFT dispersion polymerization of MMA in the presence of 10 wt % MAA. Subsequently, a certain amount of AgNO_3_ (50 mg in this case) was added to the dispersion and irradiated for another 3 h. The color of the reaction mixture changed from milky white to brown, indicating the formation of Ag nanoparticles. Figure 9a shows the TEM image of Ag composite microspheres, and a large amount of Ag nanoparticles on the particle surface were observed. The catalytic activity of the synthesized Ag composite microspheres in the reduction of methylene blue (MB) with NaBH_4_ was investigated. Figure 9b shows the catalytic properties of Ag composite microspheres under ambient conditions. The characteristic absorption peaks of MB at 614 and 664 nm were disappeared after 27 min. The color of the solution changed from blue at 0 min to transplant at 27 min, which is consistent with the data in Figure 9b. Figure 9c shows the chemical equation of the reduction of MB with NaBH_4_ in the presence of Ag composite microspheres.

## 4. Conclusions

In conclusion, we described a photoinitiated RAFT dispersion copolymerization of MMA and MAA in ethanol/water mixtures using PVP as the steric stabilizer. Within the range of concentrations of MAA that we examined (2 to 10 wt %), polymeric microspheres with narrow size distributions were obtained with similar mean diameters. The presence of a carboxyl functional RAFT agent was crucial for obtaining carboxyl-functionalized polymeric microspheres with a narrow size distribution. We also studied the effect of other reaction parameters (e.g., photoinitiator concentration, stabilizer concentration and solvent composition) on particle morphologies. Highly monodispersed carboxyl-functionalized polymeric microspheres were obtained in most cases. The number of carboxyl groups per particle was measured by titration. Finally, Ag composite microspheres were prepared by in situ reduction of AgNO_3_. The Ag composite microspheres were able to catalyze the reduction of methylene blue with NaBH_4_ as a reductant.

## Figures and Tables

**Table 1:** Synthesis and characterization data for polymeric microspheres prepared by photoinitiated RAFT dispersion polymerization of MMA with different concentrations of MAA added at the beginning of the reaction *.

Entry Number	MMA wt %	MAA wt %	HMPP wt %	BDMAT wt %	PVP wt %	Ethanol/water *w/w*	*d*_n_ µm	*CV* %
1	10	0	3	0.5	15	40/60	0.92	3.1
2	10	2	3	0.5	15	40/60	0.94	2.5
3	10	6	3	0.5	15	40/60	1.03	2.5
4	10	10	3	0.5	15	40/60	1.09	2.4

* All polymerizations were conducted at room temperature for 3 h.

**Table 2:** Synthesis and characterization data for polymeric microspheres prepared by photoinitiated RAFT dispersion polymerization of MMA at different BDMAT concentrations with either 2 or 6 wt % MAA *.

Entry Number	MMA wt %	MAA wt %	HMPP wt %	BDMAT wt %	PVP wt %	Ethanol/water *w/w*	*d*_n_ µm	*CV* %
1	10	2	3	0.25	15	40/60	0.95	4.2
2	10	2	3	0.50	15	40/60	0.94	2.1
3	10	2	3	0.75	15	40/60	0.93	2.2
4	10	6	3	0.25	15	40/60	1.21	6.5
5	10	6	3	0.50	15	40/60	1.03	2.5
6	10	6	3	0.75	15	40/60	1.01	2.1

* All polymerizations were conducted at room temperature for 3 h.

**Table 3:** Number of carboxyl groups per particle prepared by photoinitiated RAFT dispersion polymerization of MMA with different MAA concentrations.

Entry Number	MAA concentration (wt %)	Density of carboxyl groups on the particle surface (nm^−2^)	Number of carboxyl groups per particle (×10^6^)
1	0	0.45	1.2
2	2	1.48	4.1
3	6	2.19	7.3
4	10	4.29	16

## Supplementary Materials

The following are available online at www.mdpi.com/2073-4360/9/12/681/s1.
