# Supplementary material for: Carboxyl-Functionalized Polymeric Microspheres Prepared by One-Stage Photoinitiated RAFT Dispersion Polymerization
Source: Polymers (Basel). 2017 Dec 6;9(12):681. doi: 10.3390/polym9120681 (PMC6418837; doi:10.3390/polym9120681)
Supplement: Supplementary file 1 [file polymers-09-00681-s001.pdf]

SUPPORTING INFORMATION

Carboxyl-Functionalized Polymeric Microspheres prepared by One-stage Photoinitiated RAFT Dispersion Polymerization

Jianbo Tan[a, b]*, Xueliang Li[a#], Jun He[a#], Qin Xu [a], Yuxuan Zhang[a], Xiaocong Dai[a], Liangliang Yu[a], Ruiming Zeng[a], Li Zhang [a, b]*

a. *Department of Polymeric Materials and Engineering, School of Materials and Energy, Guangdong University of Technology, Guangzhou 510006, China.*

b. *Guangdong Provincial Key Laboratory of Functional Soft Condensed Matter, Guangzhou 510006, China.*

*Corresponding authors: tanjianbo@gdut.edu.cn, lizhang@gdut.edu.cn

# These authors contributed equally to this work.

ADDITIONAL RESULTS

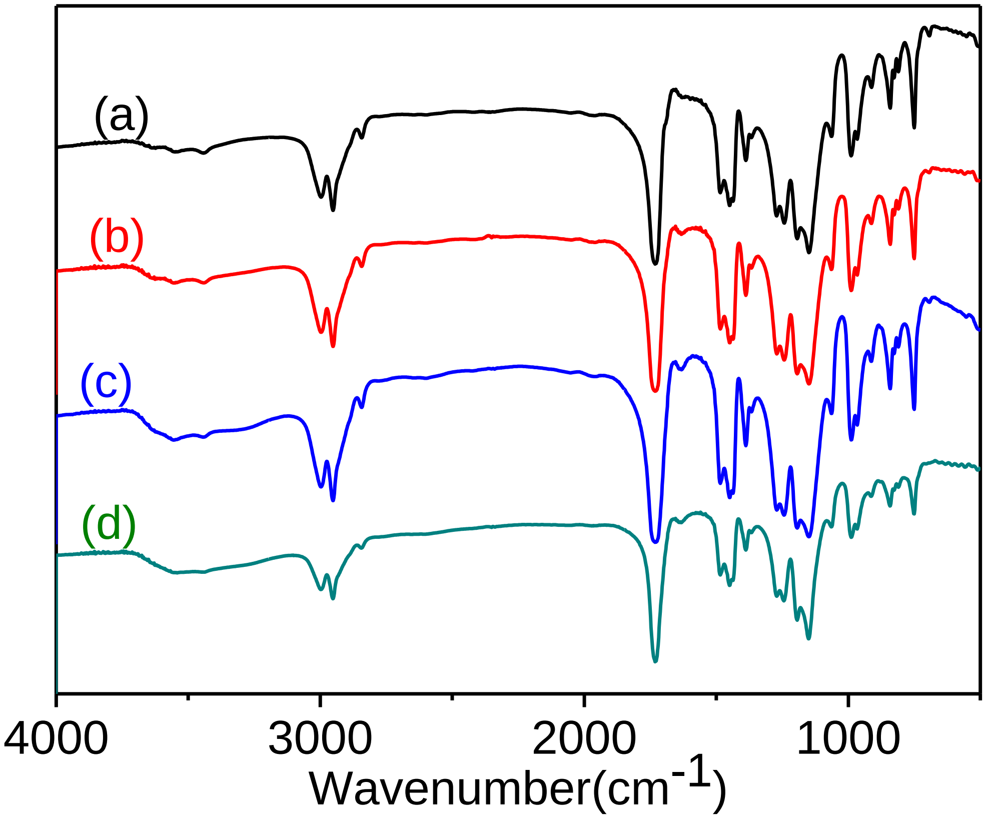

Figure S1. IR spectra of polymeric microspheres prepared via photoinitiated RAFT dispersion polymerization of MMA and MAA with different amounts of MAA: (a) 0 wt%, (b) 2 wt%, (c) 6 wt%, (d) 10 wt%.

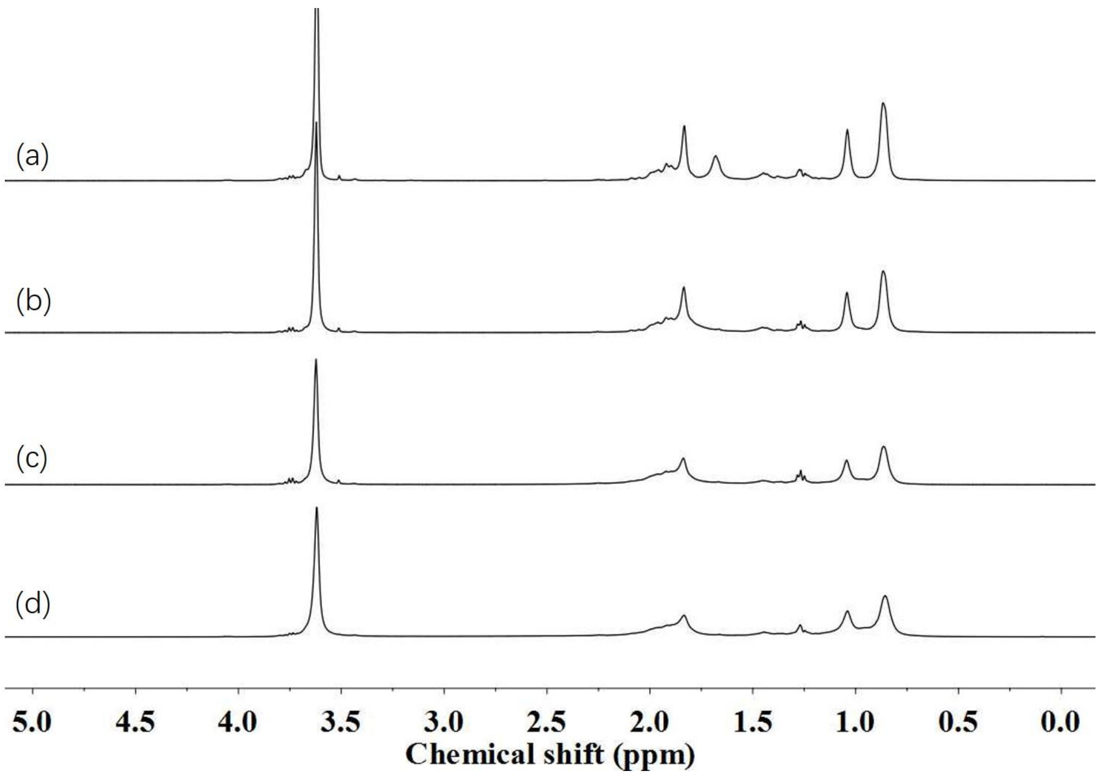

Figure S2. ^{1}H NMR spectra (in $CDCl_3$) of polymeric microspheres prepared via photoinitiated RAFT dispersion polymerization of MMA and MAA with different amounts of MAA: (a) 0 wt%, (b) 2 wt%, (c) 6 wt%, (d) 10 wt%.

Table S1. Synthesis and characterization data for polymeric microspheres prepared by photoinitiated RAFT dispersion polymerization of MMA.

Entry number	MMA wt %	MAA wt %	HMPP wt %	BDMAT wt %	PVP wt %	ethanol/water w/w	M_n (g/mol)	M_w/M_n
1	10	0	3	0.5	15	40/60	7200	3.6
2	10	2	3	0.5	15	40/60	13300	2.4
3	10	6	3	0.5	15	40/60	12600	1.9
4	10	10	3	0.5	15	40/60	15000	2.2
5	10	2	3	0.25	15	40/60	7700	3.1
6	10	2	3	0.75	15	40/60	5600	2.2
7	10	6	3	0.25	15	40/60	19900	2.0
8	10	6	3	0.75	15	40/60	6400	1.9
